# Macrophages in periapical lesions: Potential roles and future directions

**DOI:** 10.3389/fimmu.2022.949102

**Published:** 2022-09-05

**Authors:** Yao Song, Xinying Li, Dingming Huang, Hongjie Song

**Affiliations:** ^1^ Department of Stomatology, Chengdu Second People’s Hospital, Chengdu, China; ^2^ State Key Laboratory of Oral Diseases and National Clinical Research Center for Oral Diseases, West China Hospital of Stomatology, Sichuan University, Chengdu, China; ^3^ Department of Operative Dentistry and Endodontics, West China Hospital of Stomatology, Sichuan University, Chengdu, China

**Keywords:** macrophage, periapical lesion, innate immunity, macrophage polarization, oral bacteria

## Abstract

Periapical lesions are infectious diseases that occur in the apical region of teeth. They result in the destruction of alveolar bone and are usually accompanied by swelling, pain, and possible systemic impacts. A complex interaction between pathogens and the host immune system determines the development, progression, and outcome of periapical lesions. The lesions, if not treated promptly, may cause resorption of bone tissue, destruction of the periodontal ligament, and loss of the affected teeth, all of which can severely worsen the quality of life of patients, often at considerable economic cost to both patients and medical organizations. Macrophages are a group of heterogeneous cells that have many roles in the development of infections, destruction and reconstruction of bone tissues, and microbe–host interactions. However, the differential and comprehensive polarization of macrophages complicates the understanding of the regulatory mechanism of periapical lesion progression. This report provides a comprehensive review of recent advances in our knowledge of the potential role of macrophages in determining the turnover of human periapical lesions. For example, macrophage differentiation might indicate whether the lesions are stable or progressing while the extent of bacteria invasion could regulate the differentiation and function of macrophages involved in the periapical lesion. In addition, alternative strategies for the treatment of apical periodontitis are discussed.

## Introduction

Human periapical lesions (PLs) are common infectious diseases that are predominantly induced by endodontic microbial infections, especially by Gram-negative microbes, and affect the tooth and surrounding alveolar bone (1–3). PLs are an osteolytic disease that occurs in the apical region of the root (1). The prevalence of PLs in the adult population is around 6.3% worldwide; PLs reduce the lifespan and quality of life of those affected (4, 5). The treatment success rate for PLs, including those operated on by endodontic experts or specialists, is only just over 50% after 2 years’ follow-up (4, 5). Thus, despite “optimal treatment”, hundreds of thousands of teeth continue to be affected by PLs, causing symptomatic infection (5, 6). Therefore, it is essential to elucidate the mechanism of development of PLs, and to identify more effective and longer-lasting treatments (2).

Innate immunity is the first line of defence against microbial invaders and plays important roles in the development of the human immune system (7–9). Macrophages are one of the most important innate immune cells and are responsible for the recognition and clearance of exogenous pathogens in microbe-mediated infectious diseases (10–12). Macrophages play a significant role in the response to environmental challenge and in tissue remodeling after injury (9). Autocrine, endocrine, or paracrine cytokines or chemokines from different types of cells modulate the status of macrophages and influence the function and outcomes of macrophages during the progress of infectious diseases (13). In addition, cell–cell interaction through ligand–receptor binding also regulates the metabolic processes of macrophages (14, 15). Consequently, macrophages play diverse roles in regulating the inflammatory response and the reparative process in many diseases (12, 16–18). However, the precise role of macrophages in the development of PLs in humans, and the impact of macrophage dysfunction on the progress of human PLs, remain vague.

This report aims to review the clinical implications of macrophage distribution in PLs from preclinical research and laboratory studies. In addition, we discuss current experimental progress for the treatment of PLs, which might bring us new ideas in the search for new strategies in the treatment of PLs.

## The clinical implications of macrophage distribution in periapical legions

Macrophages are one of the most important functional components of the innate immune system (17). They play essential roles in maintaining physiological homeostasis and regulating the turnover of the immune responses in pathological conditions (19, 20). The spatial distribution and the polarization of macrophages might reflect the status of diseases (21, 22). We speculated that the spatial and temporal expression profile of macrophages might indicate the progression of PLs. When periapical tissues are challenged by bacterial invasion, the resident macrophages may respond to foreign material, such as bacteria and the toxins released from bacteria, and regulate the physiological and pathological process (23, 24). In the development of PLs, lipopolysaccharide (LPS) released from bacteria might bind to toll-like receptor 4 (TLR4) to initiate signal transduction and activate immune responses (25). It is believed that macrophages are a group of multifunctional and heterogeneous cells that can be divided into two subsets: M1-like macrophages and M2-like macrophages (26). M1-like macrophages, also known as classically activated macrophages or proinflammatory macrophages, are responsible for mediating and regulating the process of inflammatory responses, with the participation of T helper 1 (Th1) cells and their related cytokines. By contrast, M2-like macrophages usually exhibit anti-inflammatory roles and control the progress of efferocytosis (26–28).

In recent years, multiple groups of researchers have elucidated the distribution phenotype and preliminary function of macrophages in the development of PLs and the roles of macrophages in the different stages of PLs (29–33). Most of the observations of macrophages in PLs were from preclinical studies. Azeredo *et al. (* 34) found that cluster of differentiation 68 (CD68)-positive cells were extensively expressed in radicular cysts (RCs) and periapical granulomas (PGs). However, the number of infiltrating macrophages did not appear to differ between the two types of PL (34). These results could suggest that all macrophages found in RCs and PGs have similar expression characteristics.

Several studies that further explored the relationship between macrophage polarization and the aggressive/recovery phase of apical periodontitis (AP) have shown that the expression of M1-like or M2-like macrophages might be correlated with the pathology of PLs (31, 32). The results of quantitative immunohistochemical (IHC) studies have shown that levels of cluster of differentiation 11c (CD11c)-positive (M1-like) macrophages are higher in RCs, whereas levels of mannose receptor C type 1(MRC1)-positive (M2-like) macrophages are higher in PGs (31). The higher levels of infiltration of M1-like macrophages in RCs indicate that an aggressive state of RCs could be observed, more proinflammatory cytokines might be secreted to the lesion area, and the activated osteoclasts in the apical region of the affected teeth (33). In addition, a similar expression profile of macrophages was observed in RCs of both primary and permanent teeth (35). These studies suggest that macrophages may exhibit different expression phenotypes at different stages of PL progression. This, in turn, suggests that the expression levels of macrophages could become a detection marker for the classification of PLs, which might help in clinical diagnosis and treatment decision-making. Furthermore, several studies have mentioned a relationships between the subtypes of macrophages and the clinical symptoms of PLs. Veloso *et al. (* 36) evaluated macrophage expression of cluster of differentiation 14 (CD14), 64 (CD64), 80 (CD80), 163 (CD163), and 206 (CD206) in symptomatic apical periodontitis (SAP) and asymptomatic apical periodontitis (AAP) using flow cytometry. The expression profile of the secreted cytokines was determined using quantitative polymerase chain reaction (qPCR). The ratio of M1-like to M2-like macrophages was higher in the SAP group than in the AAP group, and macrophages from patients with SAP showed a significant reduction in CD163 expression. The expression of interleukin 6 (IL-6) and 23 (IL-23) was markedly higher in SAP patients than in AAP patients, which indicates that these cytokines might be related to the clinical presentation of PLs (36). In addition, Bracks *et al. (* 37) found that the infiltration of CD68-positive cells was accompanied by increased expression of IL-6. Džopalić *et al. (* 38) carried out multicolor flow cytometry and further explored the potential relationship between IL-6 expression and the clinical features of PLs. They demonstrated that symptomatic PLs were associated with higher levels of expression of IL-6-positive M2-like macrophages than asymptomatic PLs (38). Elucidation of the relationship between clinical symptoms and the molecular level of specific markers would help us to understand the underlying mechanisms of the presence of pain, and develop specific methods or techniques to detect and resolve the clinical symptoms of PLs.

Although numerous preclinical data have demonstrated that a large number of infiltrating M1-like macrophages might indicate a destructive state of PLs, there is no exact description of how M1-like or M2-like macrophages are distributed in PLs, which needs further biological and biochemical experimentation. In addition, owing to limitations with sample collection during the microsurgery of endodontics and the techniques used in previously mentioned studies, we could not observe the accurate distribution of macrophages and their relationships with the root, inflamed area, and surrounding bones. Therefore, further research should include more comprehensive and valid experiments and provide more solid data to elucidate the precise distribution of different subtypes of macrophages and their relationships with bone resorption area, repaired or regenerative tissues, and other types of immune cells. For example, spatial transcriptomics, single-cell sequencing, and multiple immunochemistry techniques might be applied to investigate the microenvironment of PLs, which might help us to find more specific targets of PLs. These efforts might help us to understand the failure of endodontic treatment and the determining factor in the prognosis or outcomes of PLs.

## The impact of bacteria on macrophage polarization

The presence of bacteria and other pathogens is one of the core reasons for the persistence of symptoms and other manifestations of PLs. The emergence of next-generation sequencing for micro-organism detection helped us to understand the distribution of microbial communities of APs. Hou *et al. (* 39) found that the abundance of *Porphyromonas gingivalis*, *Phocaeicola abscessus*, *Campylobacter showae*, and *Tannerella forsythia* was higher among patients with obvious symptoms than in those without, suggesting that the number and species of microbe might affect the outcome of PLs. Sun *et al. (* 40) found that the dominant bacteria isolated from extraradicular and intraradicular samples from persistent APs were quite different, which suggests that different bacteria play different roles in the pathogenesis of persistent PLs. Considering the fundamental roles of bacteria in the progression of PLs, in this section we summarize the functions of oral bacteria, such as *Enterococcus faecalis* and *P. gingivalis*, in the polarization of macrophages (41, 42).

*E. faecalis*, a Gram-positive bacterium, has been found in many bacterial studies to be closely related to the failure of the treatment of human APs and has a higher detection rate in secondary PLs than that in primary PLs (43, 44). Interestingly, several *in vitro* studies have found that *E. faecalis* plays potentially regulatory roles in the differentiation and polarization of macrophages (42, 45, 46). Park *et al. (* 46) found that *E. faecalis* affected the osteoclastogenesis of macrophages *in vitro*. In addition, *E. faecalis* activated the immune responses, promoted proinflammatory cytokine production, and maintained the phagocytic capacity of macrophages. Furthermore, the co-culture of *E. faecalis* with a human monocyte cell line suggests M2 polarization of infected macrophages, with an increase in the production of reaction oxygen species (ROS) (42). Ran *et al. (* 45) found that the presence of *E. faecalis* promoted the expression of caspase-1 and nod-like receptor family pyrin domain-containing protein 3 (NLRP3), which are key regulators of pyroptosis. *E. faecalis* could activate the pyroptosis of macrophages and induce the production and secretion of interleukin 1 beta (IL-1β) (45). These results suggest a link between *E. faecalis* and APs and endodontic failure.

*P. gingivalis* has also been recognized as an important oral pathogenic bacterium that plays crucial roles in the development of periodontitis and PLs, as well as several systematic diseases (47). *P. gingivalis* is thought to have a wider and more complicated effect on the host immune system than *E. faecalis*. *P. gingivalis*, its LPS, and its outer membrane vesicles (OMVs) might exert different immune-regulatory roles on macrophage activation (48, 49). *P. gingivalis* LPS has been reported to weakly activate the polarization of M1- or M2-like macrophages, and to induce the production of inflammatory cytokines and chemotactic cytokines, which might have a great impact on the development and outcome of PLs (50).

Despite multiple studies having preliminarily discussed the potential relationships between the presence or the invasion of different bacteria and macrophage polarization and functions, there is limited evidence demonstrating a connection between the occurrence of bacteria and their pathogenic toxins, the differentiated state of macrophages, and the destruction of alveolar bone and surrounding tissues. Therefore, future studies should focus on elucidating the precise role of macrophages in the development of human PLs, in particular the impact of secreted cytokines on the immune microenvironment and bone metabolism, and the strategies for the treatment of PLs.

## Strategies for the treatment of periapical lesions

Although root canal therapy (endodontic treatment) is considered to be the most effective treatment for PLs, and has a relatively high success rate, treatment fails in many cases, resulting in refractory inflammation and repeated infections of the affected teeth, accompanied by swelling, pain, and general infection (5). It is essential that we urgently investigate the reasons for persistent infection in alveolar bone tissues and explore new, effective strategies for the treatment of PLs, which should be specific and effective, with few side effects.

From a genetics perspective, several research groups have identified in *in vitro* and/or *in vivo* experiments molecules that play significant roles in the development of PLs and which might become therapeutic targets for the treatment of PLs in humans. Hypoxia-inducible factor 1 alpha subunit (HIF-1α) is a key molecule in the mediation of oxygen metabolism and homeostasis, and plays important roles in inflammatory reactions and bone reconstruction. Hirai *et al. (* 51) demonstrated that the application of exogenous HIF-1α could protect the progression of APs by inhibiting the secretion of proinflammatory cytokines, attenuating the M1 polarization of macrophages, and down-regulating osteoclastogenic differentiation. In another study, the regulatory role of serum amyloid A (SAA) in the development of PLs was investigated using conditional knockout (KO) mice. In this experimentally induced model of PLs, the number of infiltrating myeloid cells in the periapical region of affected teeth was lower in SAA KO mice than in wild-type (WT) mice. In addition, SAA might regulate the function of macrophages in PLs *via* toll-like receptor 2 (TLR2) and TLR4, suggesting that SAA might become a regulatory target for the treatment of PLs (52). Similarly, Wang *et al. (* 53) showed that adeno-associated virus (AAV)-mediated therapeutic methods can effectively reduce tissue destruction, attenuate the inflammatory reaction, and slow the process of alveolar bone loss. The application of *Atp6v1c1* (an AAV specific for C1 silencing) could reduce bone destruction by nearly 70%, decreasing the infiltration of inflammatory cells into the periapical region, and maintain the integrity of periodontal ligament (53). Despite the fact that more data and experiments are needed to ensure the efficiency and safety of this strategy, this study showed that AAV-mediated methods might become an effective strategy for the assistance of endodontic treatment.

As well as uncovering the underlying mechanism of PLs by molecular methods, some research groups have also tried to elucidate the potential roles of immunoregulatory agents or antioxidants in the treatment of PLs, such as melatonin, azithromycin, or metformin (54–56). Saritekin *et al. (* 54) showed that intraperitoneal injection of melatonin could attenuate the progression of PLs, leading to a reduction in inflammatory reactions and a decrease in periapical defects. This indicates that melatonin protects against the development of PLs. The application of azithromycin has also been shown to have a significant beneficial effect on APs, resulting in increased infiltration of neutrophils and M2-like macrophages. These data suggest that azithromycin might become a therapeutic option in the adjunctive treatment of PLs (55). In addition, not only could the general application of drugs help the resolution of PLs, but intracanal administration of some agents could contribute to the recovery of existing periapical lesions. Intracanal metformin could contribute to the healing of APs by suppressing monocyte recruitment and inhibiting inducible nitric oxide synthase (iNOS), which is a representative marker for M1-like macrophages (56). Although these encouraging data raised some potential strategies for the treatment of refractory PLs, more research should focus on elucidating the underlying mechanism of APs and revealing the precise function of the drug or exogenous agents on the healing of PLs. Only in this way can we improve the success rate of endodontic treatment.

## Conclusion

With more studies reporting that polarized macrophages are closely correlated with the progressive state of PLs, it is urgent for us to elucidate the precise mechanism of the regulatory roles of macrophages in the development of PLs. As research on the role of macrophages in the pathogenesis of PLs gradually progresses, we must pay more attention to the current limitations and drawbacks in the previously published studies. The predominant subtypes of macrophages at different stages of PLs, the regulatory cytokine networks that macrophages participate in, and the potential therapeutic targets on macrophage regulations need to be settled. In this way, we could understand the pathological mechanisms of PLs better, and we can find more effective strategies to improve the success rate of the treatment of PLs.

## Author contributions

HS conceived and designed this review. YS, XL, DH, and HS wrote the first draft of the manuscript and made the literature review. DH and HS critically revised the manuscript. All authors contributed to this article and approved the final version to be submitted.

## Funding

This study was supported by The Research Project of Sichuan Medical Association, No. S18012 (HS).

## Acknowledgments

We sincerely thank Doctor Liu Liu (Sichuan University) for his kind help with this manuscript.

## Conflict of interest

The authors declare that the research was conducted in the absence of any commercial or financial relationships that could be construed as a potential conflict of interest.

## Publisher’s note

All claims expressed in this article are solely those of the authors and do not necessarily represent those of their affiliated organizations, or those of the publisher, the editors and the reviewers. Any product that may be evaluated in this article, or claim that may be made by its manufacturer, is not guaranteed or endorsed by the publisher.

## References

[1] Alvarez C Monasterio G Cavalla F Cordova LA Hernandez M Heymann D. Osteoimmunology of oral and maxillofacial diseases: Translational applications based on biological mechanisms. Front Immunol (2019) 10:1664. doi: 10.3389/fimmu.2019.01664 31379856 PMC6657671

[2] Cavalla F Letra A Silva RM Garlet GP. Determinants of Periodontal/Periapical lesion stability and progression. J Dent Res (2021) 100(1):29–36. doi: 10.1177/0022034520952341 32866421

[3] Wang L Yang F Qiu Y Ye L Song D Huang D. The potential roles of T cells in periapical lesions. J Endod (2022) 48(1):70–9. doi: 10.1016/j.joen.2021.09.016 34627784

[4] Jakovljevic A Nikolic N Jacimovic J Pavlovic O Milicic B Beljic-Ivanovic K. Prevalence of apical periodontitis and conventional nonsurgical root canal treatment in general adult population: An updated systematic review and meta-analysis of cross-sectional studies published between 2012 and 2020. J Endod (2020) 46(10):1371–86. doi: 10.1016/j.joen.2020.07.007 32673634

[5] Restrepo-Restrepo FA Canas-Jimenez SJ Romero-Albarracin RD Villa-Machado PA Perez-Cano MI Tobon-Arroyave SI. Prognosis of root canal treatment in teeth with preoperative apical periodontitis: a study with cone-beam computed tomography and digital periapical radiography. Int Endod J (2019) 52(11):1533–46. doi: 10.1111/iej.13168 31211862

[6] Serefoglu B Micoogullari Kurt S Kandemir Demirci G Kaval ME Caliskan MK. A prospective cohort study evaluating the outcome of root canal retreatment in symptomatic mandibular first molars with periapical lesions. Int Endod J (2021) 54(12):2173–83. doi: 10.1111/iej.13631 34516682

[7] Baral P Udit S Chiu IM. Pain and immunity: implications for host defence. Nat Rev Immunol (2019) 19(7):433–47. doi: 10.1038/s41577-019-0147-2 PMC6700742 30874629

[8] Lemke G. How macrophages deal with death. Nat Rev Immunol (2019) 19(9):539–49. doi: 10.1038/s41577-019-0167-y PMC6733267 31019284

[9] Watanabe S Alexander M Misharin AV Budinger GRS. The role of macrophages in the resolution of inflammation. J Clin Invest (2019) 129(7):2619–28. doi: 10.1172/JCI124615 PMC6597225 31107246

[10] Ost KS Round JL. Communication between the microbiota and mammalian immunity. Annu Rev Microbiol (2018) 72:399–422. doi: 10.1146/annurev-micro-090817-062307 29927706 PMC7294967

[11] Ganal-Vonarburg SC Hornef MW Macpherson AJ. Microbial-host molecular exchange and its functional consequences in early mammalian life. Science (2020) 368(6491):604–7. doi: 10.1126/science.aba0478 32381716

[12] Zheng D Liwinski T Elinav E. Interaction between microbiota and immunity in health and disease. Cell Res (2020) 30(6):492–506. doi: 10.1038/s41422-020-0332-7 32433595 PMC7264227

[13] Abraham C Abreu MT Turner JR. Pattern recognition receptor signaling and cytokine networks in microbial defenses and regulation of intestinal barriers: Implications for inflammatory bowel disease. Gastroenterology (2022) 6:1602–16. doi: 10.1053/j.gastro.2021.12.288 PMC9112237 35149024

[14] Arteaga-Blanco LA Bou-Habib DC. The role of extracellular vesicles from human macrophages on host-pathogen interaction. Int J Mol Sci (2021) 22(19). doi: 10.3390/ijms221910262 PMC8508751 34638604

[15] Kim JM Lin C Stavre Z Greenblatt MB Shim JH. Osteoblast-osteoclast communication and bone homeostasis. Cells (2020) 9(9). doi: 10.3390/cells9092073 PMC7564526 32927921

[16] Laskin DL Sunil VR Gardner CR Laskin JD. Macrophages and tissue injury: agents of defense or destruction? Annu Rev Pharmacol Toxicol (2011) 51:267–88. doi: 10.1146/annurev.pharmtox.010909.105812 PMC3670679 20887196

[17] Michalski MN McCauley LK. Macrophages and skeletal health. Pharmacol Ther (2017) 174:43–54. doi: 10.1016/j.pharmthera.2017.02.017 28185913 PMC5429177

[18] DeNardo DG Ruffell B. Macrophages as regulators of tumour immunity and immunotherapy. Nat Rev Immunol (2019) 19(6):369–82. doi: 10.1038/s41577-019-0127-6 PMC7339861 30718830

[19] Xu H Zhang S Sathe AA Jin Z Guan J Sun W. CCR2(+) macrophages promote orthodontic tooth movement and alveolar bone remodeling. Front Immunol (2022) 13:835986. doi: 10.3389/fimmu.2022.835986 35185928 PMC8854866

[20] Wang L Sun Z Liu L Peng B. Expression of CX3CL1 and its receptor, CX3CR1, in the development of periapical lesions. Int Endod J (2014) 47(3):271–9. doi: 10.1111/iej.12143 23829599

[21] Ydens E Amann L Asselbergh B Scott CL Martens L Sichien D. Profiling peripheral nerve macrophages reveals two macrophage subsets with distinct localization, transcriptome and response to injury. Nat Neurosci (2020) 23(5):676–89. doi: 10.1038/s41593-020-0618-6 PMC7611025 32284604

[22] Guilliams M Bonnardel J Haest B Vanderborght B Wagner C Remmerie A. Spatial proteogenomics reveals distinct and evolutionarily conserved hepatic macrophage niches. Cell (2022) 185(2):379–396 e338. doi: 10.1016/j.cell.2021.12.018 35021063 PMC8809252

[23] Goldman E Reich E Roshihotzki B Saketkhou M Wald S Goldstein A. A mouse model for studying the development of apical periodontitis with age. Cells (2021) 10(3). doi: 10.3390/cells10030671 PMC8002842 33802950

[24] Ricucci D Rocas IN Hernandez S Siqueira JF Jr. "True" versus "Bay" apical cysts: Clinical, radiographic, histopathologic, and histobacteriologic features. J Endod (2020) 46(9):1217–27. doi: 10.1016/j.joen.2020.05.025 32544498

[25] Latz E Visintin A Lien E Fitzgerald KA Monks BG Kurt-Jones EA. Lipopolysaccharide rapidly traffics to and from the golgi apparatus with the toll-like receptor 4-MD-2-CD14 complex in a process that is distinct from the initiation of signal transduction. J Biol Chem (2002) 277(49):47834–43. doi: 10.1074/jbc.M207873200 12324469

[26] Silva LM Doyle AD Greenwell-Wild T Dutzan N Tran CL Abusleme L. Fibrin is a critical regulator of neutrophil effector function at the oral mucosal barrier. Science (2021) 374(6575):eabl5450. doi: 10.1126/science.abl5450 34941394 PMC11960105

[27] Michalski MN Koh AJ Weidner S Roca H McCauley LK. Modulation of osteoblastic cell efferocytosis by bone marrow macrophages. J Cell Biochem (2016) 117(12):2697–706. doi: 10.1002/jcb.25567 PMC5055427 27061191

[28] Meyle J Dommisch H Groeger S Giacaman RA Costalonga M Herzberg M. The innate host response in caries and periodontitis. J Clin Periodontol (2017) 44(12):1215–25. doi: 10.1111/jcpe.12781 28727164

[29] Kopp W Schwarting R. Differentiation of T lymphocyte subpopulations, macrophages, and HLA-DR-restricted cells of apical granulation tissue. J Endod (1989) 15(2):72–5. doi: 10.1016/S0099-2399(89)80111-6 2607272

[30] Akamine A Anan H Hamachi T Maeda K. A histochemical study of the behavior of macrophages during experimental apical periodontitis in rats. J Endod (1994) 20(10):474–8. doi: 10.1016/S0099-2399(06)80042-7 7714418

[31] Weber M Schlittenbauer T Moebius P Buttner-Herold M Ries J Preidl R. Macrophage polarization differs between apical granulomas, radicular cysts, and dentigerous cysts. Clin Oral Investig (2018) 22(1):385–94. doi: 10.1007/s00784-017-2123-1 28501945

[32] Franca GM Carmo AFD Costa Neto H Andrade A Lima KC Galvao HC. Macrophages subpopulations in chronic periapical lesions according to clinical and morphological aspects. Braz Oral Res (2019) 33:e047. doi: 10.1590/1807-3107bor-2019.vol33.0047 31141038

[33] Weber M Ries J Buttner-Herold M Geppert CI Kesting M Wehrhan F. Differences in inflammation and bone resorption between apical granulomas, radicular cysts, and dentigerous cysts. J Endod (2019) 45(10):1200–8. doi: 10.1016/j.joen.2019.06.014 31400944

[34] Azeredo SV Brasil SC Antunes H Marques FV Pires FR Armada L. Distribution of macrophages and plasma cells in apical periodontitis and their relationship with clinical and image data. J Clin Exp Dent (2017) 9(9):e1060–5. doi: 10.4317/jced.53758 PMC5650206 29075406

[35] Bertasso AS Leon JE Silva RAB Silva LAB de Queiroz AM Pucinelli CM. Immunophenotypic quantification of M1 and M2 macrophage polarization in radicular cysts of primary and permanent teeth. Int Endod J (2020) 53(5):627–35. doi: 10.1111/iej.13257 31845371

[36] Veloso P Fernandez A Terraza-Aguirre C Alvarez C Vernal R Escobar A. Macrophages skew towards M1 profile through reduced CD163 expression in symptomatic apical periodontitis. Clin Oral Investig (2020) 24(12):4571–81. doi: 10.1007/s00784-020-03324-2 32444919

[37] Bracks IV Armada L Gonçalves LS Pires FR. Distribution of mast cells and macrophages and expression of interleukin-6 in periapical cysts. J Endod (2014) 40(1):63–8. doi: 10.1016/j.joen.2013.09.037 24331993

[38] Džopalić T Tomić S Bekić M Vučević D Mihajlović D Eraković M. Ex vivo study of IL-6 expression and function in immune cell subsets from human periapical lesions. Int Endod J (2022) 55(5):480–94. doi: 10.1111/iej.13704 35150455

[39] Hou Y Wang L Zhang L Tan X Huang D Song D. Potential relationship between clinical symptoms and the root canal microbiomes of root filled teeth based on the next-generation sequencing. Int Endod J (2022) 55(1):18–29. doi: 10.1111/iej.13640 34592001

[40] Sun X Yang Z Nie Y Hou B. Microbial communities in the extraradicular and intraradicular infections associated with persistent apical periodontitis. Front Cell Infect Microbiol (2021) 11:798367. doi: 10.3389/fcimb.2021.798367 35096647 PMC8791237

[41] Meka SRK Younis T Reich E Elayyan J Kumar A Merquiol E. TNFalpha expression by porphyromonas gingivalis-stimulated macrophages relies on Sirt1 cleavage. J Periodontal Res (2021) 56(3):535–46. doi: 10.1111/jre.12853 33559894

[42] Polak D Yaya A Levy DH Metzger Z Abramovitz I. Enterococcus faecalis sustained infection induces macrophage pro-resolution polarization. Int Endod J (2021) 54(10):1840–9. doi: 10.1111/iej.13574 34013580

[43] Stuart CH Schwartz SA Beeson TJ Owatz CB. Enterococcus faecalis: its role in root canal treatment failure and current concepts in retreatment. J Endod (2006) 32(2):93–8. doi: 10.1016/j.joen.2005.10.049 16427453

[44] Bouillaguet S Manoil D Girard M Louis J Gaïa N Leo S. Root microbiota in primary and secondary apical periodontitis. ront Microbiol (2018) 9:2374. doi: 10.3389/fmicb.2018.02374 PMC6189451 30356779

[45] Ran S Huang J Liu B Gu S Jiang W Liang J. Enterococcus faecalis activates NLRP3 inflammasomes leading to increased interleukin-1 beta secretion and pyroptosis of THP-1 macrophages. Microb Pathog (2021) 154:104761. doi: 10.1016/j.micpath.2021.104761 33524566

[46] Park OJ Yang J Kim J Yun CH Han SH. Enterococcus faecalis attenuates the differentiation of macrophages into osteoclasts. J Endod (2015) 41(5):658–62. doi: 10.1016/j.joen.2014.12.015 25649294

[47] Mei F Xie M Huang X Long Y Lu X Wang X. Porphyromonas gingivalis and its systemic impact: Current status. Pathogens (2020) 9(11). doi: 10.3390/pathogens9110944 PMC7696708 33202751

[48] Fleetwood AJ Lee MKS Singleton W Achuthan A Lee MC O'Brien-Simpson NM. Metabolic remodeling, inflammasome activation, and pyroptosis in macrophages stimulated by porphyromonas gingivalis and its outer membrane vesicles. Front Cell Infect Microbiol (2017) 7:351. doi: 10.3389/fcimb.2017.00351 28824884 PMC5543041

[49] Yu S Ding L Liang D Luo L. Porphyromonas gingivalis inhibits M2 activation of macrophages by suppressing alpha-ketoglutarate production in mice. Mol Oral Microbiol (2018) 33(5):388–95. doi: 10.1111/omi.12241 30007055

[50] Holden JA Attard TJ Laughton KM Mansell A O'Brien-Simpson NM Reynolds EC. Porphyromonas gingivalis lipopolysaccharide weakly activates M1 and M2 polarized mouse macrophages but induces inflammatory cytokines. Infect Immun (2014) 82(10):4190–203. doi: 10.1128/IAI.02325-14 PMC4187848 25047849

[51] Hirai K Furusho H Hirota K Sasaki H. Activation of hypoxia-inducible factor 1 attenuates periapical inflammation and bone loss. Int J Oral Sci (2018) 10(2):12. doi: 10.1038/s41368-018-0015-0 29654284 PMC5966812

[52] Hirai K Furusho H Kawashima N Xu S de Beer MC Battaglino R. Serum amyloid a contributes to chronic apical periodontitis *via* TLR2 and TLR4. J Dent Res (2019) 98(1):117–25. doi: 10.1177/0022034518796456 PMC6304714 30189157

[53] Wang Y Chen W Hao L McVicar A Wu J Gao N. C1 silencing attenuates inflammation and alveolar bone resorption in endodontic disease. J Endod (2019) 45(7):898–906. doi: 10.1016/j.joen.2019.02.024 31104818 PMC7988436

[54] Saritekin E Ureyen Kaya B Asci H Ozmen O. Anti-inflammatory and antiresorptive functions of melatonin on experimentally induced periapical lesions. Int Endod J (2019) 52(10):1466–78. doi: 10.1111/iej.13138 31063611

[55] Andrada AC Azuma MM Furusho H Hirai K Xu S White RR. Immunomodulation mediated by azithromycin in experimental periapical inflammation. J Endod (2020) 46(11):1648–54. doi: 10.1016/j.joen.2020.07.028 PMC7862415 32763436

[56] Wang HW Lai EH Yang CN Lin SK Hong CY Yang H. Intracanal metformin promotes healing of apical periodontitis *via* suppressing inducible nitric oxide synthase expression and monocyte recruitment. J Endod (2020) 46(1):65–73. doi: 10.1016/j.joen.2019.10.001 31753516

